# Evaluating the pathogenic and immunological effects of ds-GFP as a control in *in vivo* RNA interference studies of *Schistosoma japonicum*

**DOI:** 10.1051/parasite/2025003

**Published:** 2025-02-26

**Authors:** Lei Xiu, Xiaoling Wang, Shaoyun Cheng, Wanling Liu, Lu Wang, Jiaqi Li, Jinrui Zhang, Yaping Xuan, Wei Hu

**Affiliations:** 1 State Key Laboratory of Reproductive Regulation and Breeding of Grassland Livestock, Institutes of Biomedical Sciences, School of Life Sciences, Inner Mongolia University Hohhot 010030 China; 2 State Key Laboratory of Genetic Engineering, School of Life Sciences, Fudan University Shanghai 200438 China; 3 Department of Infectious Diseases, Shanghai Key Laboratory of Infectious Diseases and Biosafety Emergency Response, National Medical Center for Infectious Diseases, Huashan Hospital, Fudan University Shanghai 200438 China

**Keywords:** *Schistosoma japonicum*, Double-stranded RNA, RNA interference, Immune responses, ds-GFP

## Abstract

Schistosomiasis affects over 250 million people in 78 countries. Despite praziquantel as the primary treatment, concerns about resistance in schistosomes underscore the need for alternative therapies. The success of RNA interference (RNAi) in schistosomes shows promise for identifying potential drug targets to facilitate drug discovery. Meanwhile, double-stranded RNA (dsRNA) is commonly used in functional gene analysis *via* RNAi, with double-stranded green fluorescent protein (ds-GFP) widely employed as a control in schistosome-related studies. However, the potential for off-target effects of dsRNAs in various biological systems raises concerns about the reliability of conventional controls in schistosome RNAi experiments. Therefore, this study aims to evaluate the safety and suitability of ds-GFP as an RNAi negative control in *Schistosoma japonicum*. Our data indicate that ds-GFP is innocuous and exerts no discernible impact on the host’s physiology and immune responses. Comprehensive evaluations conducted in mice showed no significant alterations in body and organ weights. While a splenic immune response was observed, histopathological examinations of multiple organs confirmed the absence of significant lesions following ds-GFP treatment. Additionally, *S. japonicum* morphology, reproductive capacity, and host responses to parasite eggs showed no significant variations. Taken together, these findings bolster the endorsement of ds-GFP as an appropriate negative control in *S. japonicum* RNAi experiments, offering reliable outcomes crucial for advancing research on schistosomiasis and related parasitic diseases.

## Abstract (fr)

La schistosomiase touche plus de 250 millions de personnes dans 78 pays. Bien que le praziquantel soit le traitement principal, les inquiétudes concernant la résistance des schistosomes soulignent la nécessité de thérapies alternatives. Le succès de l’interférence ARN (ARNi) chez les schistosomes est prometteur pour identifier des cibles médicamenteuses potentielles afin de faciliter la découverte de médicaments. Parallèlement, l’ARN double brin (ARNdb) est couramment utilisé dans l’analyse fonctionnelle des gènes *via* l’ARNi, la protéine fluorescente verte double brin (PFV-db) étant largement utilisée comme contrôle dans les études liées aux schistosomes. Cependant, le potentiel d’effets hors cible des ARNdb dans divers systèmes biologiques soulève des inquiétudes quant à la fiabilité des contrôles conventionnels dans les expériences d’ARNi sur les schistosomes. Par conséquent, cette étude vise à évaluer la sécurité et l’adéquation de la PFV-db comme contrôle négatif de l’ARN interférent chez *Schistosoma japonicum*. Nos données indiquent que la PFV-db est inoffensive et n’exerce aucun impact perceptible sur la physiologie et les réponses immunitaires de l’hôte. Des évaluations complètes menées sur des souris n’ont montré aucune altération significative du poids du corps et des organes. Bien qu’une réponse immunitaire splénique ait été observée, des examens histopathologiques de plusieurs organes ont confirmé l’absence de lésions significatives après le traitement par la PFV-db. De plus, la morphologie de *S. japonicum*, la capacité de reproduction et les réponses de l’hôte aux œufs du parasite n’ont montré aucune variation significative. Prises ensemble, ces découvertes renforcent l’approbation de la PFV-db comme contrôle négatif approprié dans les expériences d’ARNi sur *S. japonicum*, offrant des résultats fiables essentiels pour faire progresser la recherche sur la schistosomiase et les maladies parasitaires associées.

## Introduction

Schistosomiasis is a neglected tropical disease caused by parasitic trematodes of the genus *Schistosoma*, affecting both humans and animals. Utilizing modern molecular biology techniques, transcriptome analyses of various developmental stages of *S. japonicum* and dynamic transcriptome analyses from cercariae to egg-laying stages have been conducted [11, 15, 21]. These studies provide novel insights for screening effective anti-schistosome vaccine candidates and drug targets. Furthermore, in-depth exploration of molecules with potential key functions and elucidation of their roles in schistosome development, evolution, and host interactions highlight RNA interference (RNAi) as a valuable research tool. Double-stranded RNA (dsRNA) as a trigger for RNAi is a well-established technical approach for functional gene analyses in many organisms, including parasites [1, 8, 17, 20]. In 2003, the initial utilization of RNAi was reported in the parasitic flatworm *S. mansoni* [5, 18]. Since then, RNAi has emerged as an indispensable tool in reverse genetics for this and other parasites, demonstrating its significant value in scientific research. In the absence of an *in vitro* culture system supporting sexual maturity and organ function in *S. japonicum*, traditional RNAi methods for gene expression suppression were deemed unsuitable. To overcome this limitation, we previously pioneered a method for RNAi-mediated gene expression suppression throughout the complete life-cycle of *S. japonicum* within experimentally infected mouse hosts [14].

RNA interference (RNAi) is a highly conserved cellular process that controls the expression of foreign genes and chromosomal function in a wide variety of animals [1, 10]. Initiated by double-stranded RNA (dsRNA), Dicer and Drosha enzymes process it into short interfering RNAs (siRNA) of 19–23 nucleotides. These siRNAs, in conjunction with argonaute proteins, assemble into the RNA-induced silencing complex (RISC), which selectively binds to specific RNA targets, leading to their degradation [16]. RNAi has emerged as a preferred tool in parasite research for investigating gene function, capitalizing on the intrinsic RNAi machinery of these organisms. This method holds particular significance in parasitology, where, unlike model organisms, gene knock-out techniques have yet to be established [7]. Comprehensive transcriptome and genome sequencing data for schistosomes has made it possible to identify putative gene targets and devise novel tactics. Since the introduction of transient RNAi in 2003, utilizing both long and short-interfering dsRNA, it has proven to be a straightforward and essential tool for studying gene function in schistosomes and other parasites [5, 18, 20].

Under *in vitro* circumstances, *S. japonicum* cannot achieve sexual maturity, and reproductive organs of sexually mature parasites degenerate. Consequently, utilizing RNAi methods for gene expression suppression to validate the corresponding phenotypes of functional genes is not suitable within an *in vitro* worm culture model system. We have solved this problem by using a new method that uses RNAi to decrease gene expression in *S. japonicum* throughout its complete life cycle in mouse hosts that were experimentally infected [14]. Meanwhile, adapting the approach to the parasite’s complicated life phases within the mammalian host requires a detailed understanding of host-parasite interaction dynamics. Understanding the host immunological response, cellular absorption pathway, and the stability of dsRNA *in vivo* is critical for the successful application of RNAi techniques.

Nevertheless, the established potential for off-target effects of dsRNAs in diverse biological systems raises concerns about the reliability of conventional controls in *S. japonicum* RNAi research [9, 17, 22]. These controls frequently contain “scrambled” sequences of the target-oriented dsRNA, as well as non-schistosome dsRNAs targeting genes such as *gfp*, *mcherry*, and luciferase [16]. While these sequences are initially believed to have no nonspecific effects, observed concerns, such as altered expression of off-target genes, propose the creation of siRNAs that match non-homologous sequences. This underscores the need for a thorough reevaluation of these controls to prove the accuracy and specificity of RNAi experiments in schistosomes.

Therefore, the primary goal of our study was to reevaluate the safety and usefulness of double-stranded green fluorescent protein (ds-GFP), which is routinely employed as RNAi control in *S. japonicum*. This evaluation was critical for establishing the consistency of experimental results in RNAi research with *S. japonicum*. Our study involving *in vivo* experiments with mice infected by *S. japonicum* cercariae showed that ds-GFP treatment did not cause significant body weight loss or immune cell profile changes, despite a splenic immune response indicated by elevated cytokines at 28 days post-infection. Furthermore, ds-GFP did not induce organ damage or adversely affect the growth, development, or reproductive capacity of *S. japonicum*. These results support the safety and reliability of ds-GFP as a control in RNAi studies related to schistosomiasis and similar parasitic diseases.

## Materials and methods

### Ethics statement

All animal procedures in this study were conducted in accordance with the animal husbandry guidelines of Fudan University. The design and implementation of all animal experiments was in accordance with the 3R requirements of experimental animal ethics (Replacement, Reduction, and Refinement) and approved by the Ethical Committee and the Experimental Animal Committee of Fudan University, with ethical clearance numbers 2021JS0044.

### Parasite material

All parasite material was from an Anhui isolate of *S. japonicum* maintained in the National Institute of Parasitic Diseases, Chinese Center for Diseases Control and Prevention, Shanghai.

### Animals

Six- to eight-week-old female Kunming or C57BL/6 mice were purchased from Shanghai JieSiJie Laboratory Animal Co., Ltd. (Shanghai, China) (permit number: SCXKZ Shanghai 2018–0004). All the mice were housed under controlled environmental conditions of temperature (25 ± 2 °C) with a normal day/night cycle and humidity (55–60%) and were acclimatized for a period of 7 days maintained on a basal diet and water *ad libitum* prior to the formal experiment.

### Synthesis of double-stranded RNA

The primers were meticulously designed, and their sequences are detailed in Supplementary Table 1. Subsequent to the polymerase chain reaction (PCR), DNA sequencing was employed to validate the amplified product. The confirmed product was extracted and recovered from agarose gels, and its identity was verified by Tsingke Biotechnology Co., Ltd (Shanghai, China). The recovered material underwent preparation and purification utilizing a MEGAscript™ T7 High Yield Transcription Kit (Invitrogen™, Waltham, MA, USA; Cat #: AM1334). The size and integrity of the resulting dsRNA, 451 bp in length for GFP, were confirmed through 1% agarose gel electrophoresis. The synthesized dsRNA was then aliquoted and securely stored at -80 °C for subsequent experimental applications.

### RNA interference of *Schistosoma japonicum in vivo* and sample collection

Each mouse was infected with 60 ± 2 cercariae percutaneously and randomly divided into two groups, with four mice per group. NaCl and green fluorescent protein (GFP) dsRNA were injected into the tail vein for interference at 1, 6, 10, 14, 18, 22, and 26 dpi [14], and the influence of *S. japonicum* on the growth and development of the worms in mice was observed by harvesting worms at 28 dpi. For the egg-laying model, eight mice were randomly divided into two groups, the NaCl group and the GFP control group, 40 cercariae were used per mouse. Mice were euthanized at 42 days and the liver was collected to detect changes in the number of hepatic eggs [6].

### Tissue harvest

The peripheral blood and serum were collected from the fundus venous plexus of mice for blood routine test and Luminex liquid suspension chip detection at 0, 14, 21, and 28 dpi. The abdominal cavity was opened, and fresh liver, spleen, heart, kidney, small intestine, and lung tissues were washed in cold phosphate-buffered saline (PBS), after weighing and part of the spleen tissues were taken for FCM assay, while the rest of the tissues were stored at − 80 °C for RT-qPCR assay. The tissues were fixed in 4% paraformaldehyde for more than 24 h for hematoxylin–eosin staining.

### Hematoxylin–eosin (HE) staining

Tissues fixed in 4% paraformaldehyde fixative for 24 h were paraffin embedded, sectioned, stained with hematoxylin and eosin stain, and sealed with neutral resin and observed under the microscope. The nucleus became blue, while the cytoplasm became red or pink.

### Masson’s trichrome staining

The harvested liver tissues were fixed in formalin and then embedded in paraffin blocks. Thin sections were obtained using a microtome, deparaffinized, and rehydrated. The staining process included nuclei staining with Weigert’s iron hematoxylin, differentiation with Biebrich scarlet-acid fuchsin, color contrast enhancement with phosphomolybdic-phosphotungstic acid, and selective staining of collagen fibers with aniline blue. Excess stain was removed using acetic acid. After dehydration, clearing, and mounting, the stained liver tissue sections were examined under a light microscope.

### Luminex liquid suspension chip detection

Luminex liquid suspension chip detection was performed by Wayen Biotechnologies (Shanghai, China). A Bio-Plex Pro Mouse Cytokine Grp I Panel 23-plex kit (Bio-Rad, Hercules, CA, USA; Cat #: M60009RDPD) was used in accordance with the manufacturer’s instructions. In brief, serum was incubated in 96-well plates embedded with microbeads for 30 min, and then incubated with detection antibody for 30 min. Subsequently, streptavidin-PE was added into each well for 10 min, and values were read using the Bio-Plex MAGPIX System (Bio-Rad).

### Biochemical indicator detection

The blood samples of mice from each time point were collected and prepared for anticoagulant blood. The levels of white blood cells (WBC), lymphocytes (LYM), granulocytes (GRAN), monocytes (MONO), red blood cells (RBC), hemoglobin (HGB) and platelets (PLT) were determined using an automatic blood analyzer (Servicebio, Wuhan, China).

### Flow cytometry

Flow cytometry was employed to analyze cellular populations and assess immune cell responses in the spleen following dsRNA treatment. Harvested spleens from mice were treated with dsRNA or control solutions and single-cell suspensions were prepared by mechanically dissociating the spleens and passing the cells through a cell strainer. Single-cell suspensions were stained with a panel of surface monoclonal antibodies (mAbs) in FACS buffer (PBS containing 2 mM EDTA and 0.5% BSA) for 30 min on ice, including fluorescein isothiocyanate (FITC)–conjugated anti-mouse CD11b (eBioscience, San Diego, CA, USA; Cat #: 11-0112-82) and Pe-cyanine7–conjugated anti-mouse CD3 (eBioscience, Cat #: 25-0032-82). Finally, after two washes, all cells were resuspended in PBS. Flow cytometric analysis was performed on a five-laser BD LSRFortessa (BD Biosciences, Franklin Lakes, NJ, USA).

### Worm length analysis

To assess parasite growth, the worms treated with dsRNA were fixed using AFA (95% alcohol, 3% formaldehyde, and 2% glacial acetic acid) and subsequently photographed under a Nikon SMZ445 (Nikon, Tokyo, Japan) dissecting microscope. Digital micrographs capturing worm length and diameter were then subjected to quantitative analysis using ImageJ software (Version 1.54, https://imagej.nih.gov/ij/) and GraphPad Prism software (Version 9.3.0, https://www.graphpad.com/).

### Liver egg burden assessment

For the quantification of egg burden, liver tissue samples (~0.5 g) were individually weighed and homogenized to create homogenate suspensions. These suspensions were then subjected to digestion with a 10 mL 5% NaOH solution overnight at 37 °C. The subsequent degree of egg burden was determined through microscopic observation. Specifically, the number of eggs present in each liver homogenate was counted on 10 slides.

### RNA sequencing

To isolate the total RNA from the spleen or worms, we used an RNeasy Plus Universal Mini Kit (QIAGEN^®^ Hilden, Germany; Cat #: 73404). RNA quality was assessed by 1% agarose gel electrophoresis and a NanoPhotometer spectrophotometer (Implen, Munich, Germany). RNA integrity was assessed using an RNA Nano 6000 Assay Kit of the Bioanalyzer 2100 system (Agilent Technologies, Santa Clara, CA, USA). RNA-Seq libraries were generated with NEBNext^®^ Ultra™ II Directional RNA Library Prep with Sample Purification Beads (New England Biolabs, Ipswich, MA, USA; Cat #: E7765L), according to the manufacturer’s protocol. After cluster generation on a cBot Cluster Generation System using a TruSeq PE Cluster Kit v3-cBot-HS (Illumina, San Diego, CA, USA), the libraries were sequenced on an Illumina Novaseq platform (Novogene, Tianjin, China) with paired-end 150 bp.

### Differential expression gene analysis and functional enrichment analysis

Sequencing quality was evaluated by FastQC software (http://www.bioinformatics.babraham.ac.uk/projects/fastqc/). Poor-quality reads and adaptors were trimmed by Trimmomatic software (released version 0.22, http://www.bioinformatics.babraham.ac.uk/projects/fastqc/), and only reads longer than 50 bp were used for further analysis. The high-quality reads were mapped to the mouse genome (mouse BALB/cJ) downloaded from the Ensembl database or chromosome-level *S. japonicum* reference genome (SjV3). The HTseq [4] was used to quantify gene expression, and the R DEseq2 package [3] was employed for differential expression analysis. Only genes with false discovery rate (FDR) adjusted *p* < 0.05 and the absolute value of log 2 (fold change) ≥ 1 were considered as differential expression genes (DEGs). Functional enrichment of Gene Ontology (GO) terms analyses of DEGs were conducted by the R Cluster Profiler package [23] with FDR correction. Significantly enriched GO terms were identified with corrected *p* < 0.05.

### qPCR analysis

TRIzol reagent (Invitrogen™, Cat #: 15596026CN) was used to extract total RNA from mice and reverse transcription was performed using a cDNA reverse transcription kit (Takara Bio, Otsu, Shiga, Japan; Cat #: 6210B). The cDNA was used as a template for quantitative polymerase chain reaction (qPCR) with the Hieff^®^ qPCR SYBR Green Master Mix (Yeasen Biotechnology, Shanghai, China; Cat #: 11201ES) and 0.2 μM forward and reverse primers. The amplification conditions were 94 °C for 5 min, followed by 38 cycles of 94 °C for 30 s, 55 °C for 30 s, 72 °C for 50 s, and 72 °C for 10 min. Primer sequences are listed in Supplementary Table 1. Gene expression was calculated using the 2^(−ΔΔCT)^ method, as previously described [14], and GAPDH as an endogenous control to normalize mRNA levels [12].

### Statistical analysis

Data were analyzed using software program GraphPad Prism (Version 9.3.0, https://www.graphpad.com/). Values are expressed as mean ± SEM. Unpaired *t*-test or one-way analysis of variance (ANOVA) was used for comparison of two group or multiple groups. A non-parametric test analysis (Kruskal–Wallis or Mann–Whitney) was used for comparison groups with few samples. *, *p* < 0.05; **, *p* < 0.01; ***, *p* < 0.001; ****, *p* < 0.0001.

## Results

### Effects of ds-GFP on body weight and cytokines in mice

**Figure 1:**
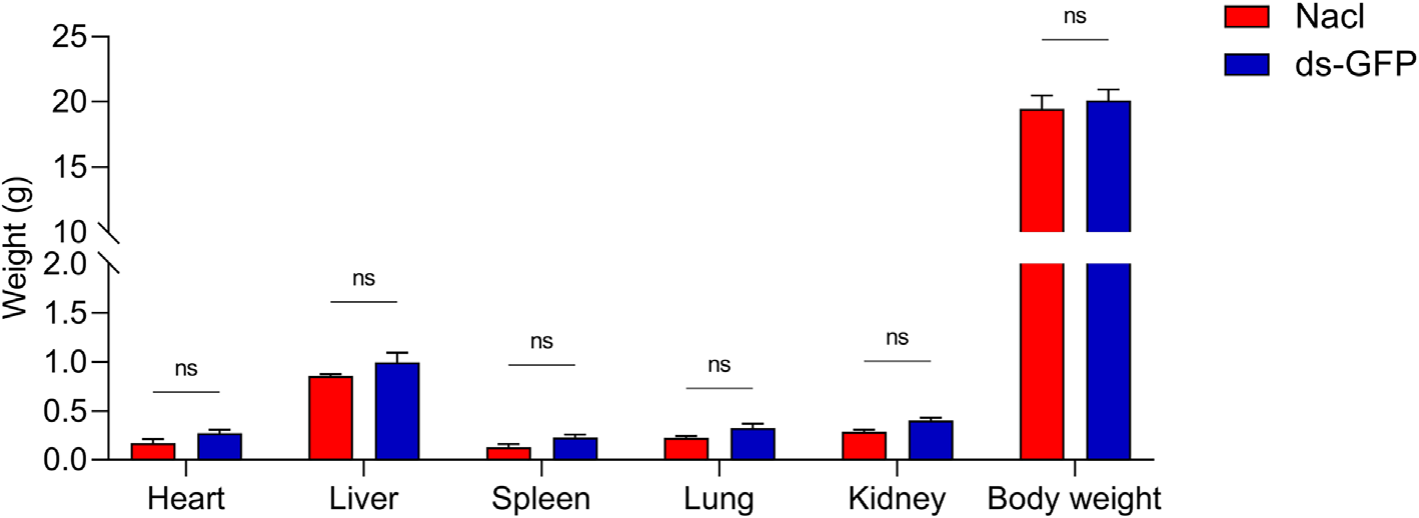
Effects of ds-RNA on body and organ weights in mice. Eight mice were randomly divided into the NaCl group and the ds-GFP group, with each mouse being infected percutaneously with 60 ± 2 cercariae. The mice were euthanized 28 days after NaCl or ds-GFP treatment and the body and organs were weighed. ns, *p* > 0.05 by Two-way ANOVA with Šídák’s multiple comparisons test; *n* = 4. Error bars represent the SEM.

To investigate the effect of ds-GFP on body and organ weights, C57BL/6 J mice treated with ds-GFP or NaCl were euthanized and the body weights and organ weights were measured. There was no significant difference in body weight between the ds-GFP-treated and NaCl-treated group over the course of 28 days (Fig. 1). Furthermore, the weights of five organs (heart, lung, liver, kidney, and spleen) did not significantly change in the ds-GFP-treated group and NaCl-treated group (Fig. 1). Moreover, food and drink intake were maintained at the same levels between ds-GFP-treated mice and NaCl-treated mice (data not shown). Therefore, the above results suggest that ds-RNA dose not induced any significant decrease on body and organ weights of mice.

**Figure 2:**
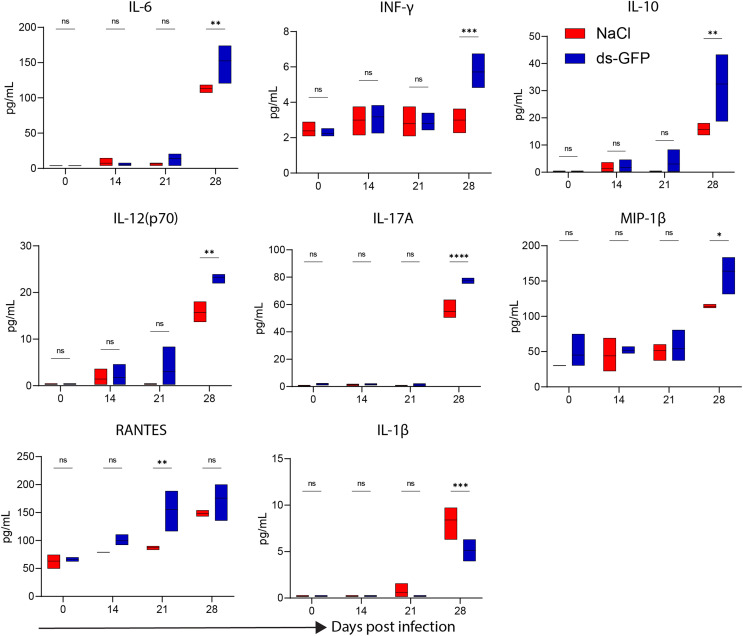
Analysis of cytokine/chemokine levels in serum of ds-RNA treated mice. Eight mice were randomly divided into the NaCl group and the ds-GFP group, with each mouse being infected percutaneously with 60 ± 2 cercariae. The mice were euthanized 28 days after NaCl or ds-GFP treatment and the serum were collected. Levels of cytokines or chemokines in the serum were detected using the Luminex liquid suspension chip assay. ****p < 0.0001, ***p < 0.001, **p < 0.01, *p < 0.05, ns *p* > 0.05 by unpaired *t*-test; *n* = 4. Error bars represent the SEM.

**Figure S1.**
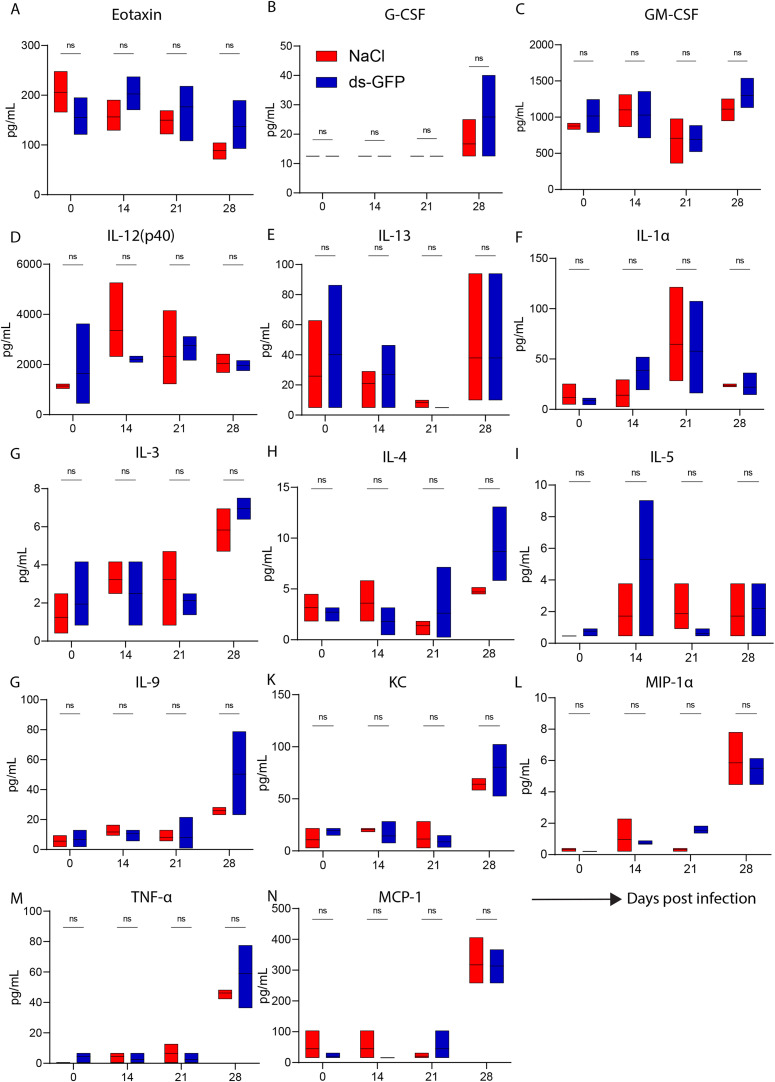
Analysis of cytokine/chemokine levels in serum of ds-RNA treated mice. Eight mice were randomly divided into the NaCl group and the ds-GFP group, with each mouse being infected percutaneously with 60 ± 2 cercariae. The mice were euthanized at a specific time after NaCl or ds-GFP treatment and sera were collected. Levels of cytokines or chemokines in the serum were detected using the Luminex liquid suspension chip assay. ns *p* > 0.05 by unpaired *t*-test. *n* = 4. Error bars represent the SEM.

To evaluate the immunological response of ds-GFP in mice, changes in serum cytokines and chemokines of ds-GFP- or NaCl-treated mice were investigated. Using the multiplex cytokine bead array assay, we analyzed 22 cytokines/chemokines in serum samples from ds-GFP- or NaCl-treated mice at days 0, 14, 21, and 28 post-injection. On 0, 14, and 21 days post-injection (dpi), there were no differences in the protein levels of most of the cytokines/chemokines examined between the groups, except for RANTES, which was upregulated in the ds-GFP-treated mice on day 21 post-injection (Fig. 2 and Fig. S1). On day 28 post-injection, ds-GFP-treated mice showed significantly increased concentrations of the pro-inflammatory cytokines interleukin-6 (IL-6), IL-17A, IL-12(p70), interferon-γ (IFN-γ), MIP-β and anti-inflammatory cytokines IL-10 compared to NaCl-treated mice (Fig. 2). These results showed that ds-GFP treatment caused a slight increase in cytokines, particularly at 28 dpi.

**Figure 3:**
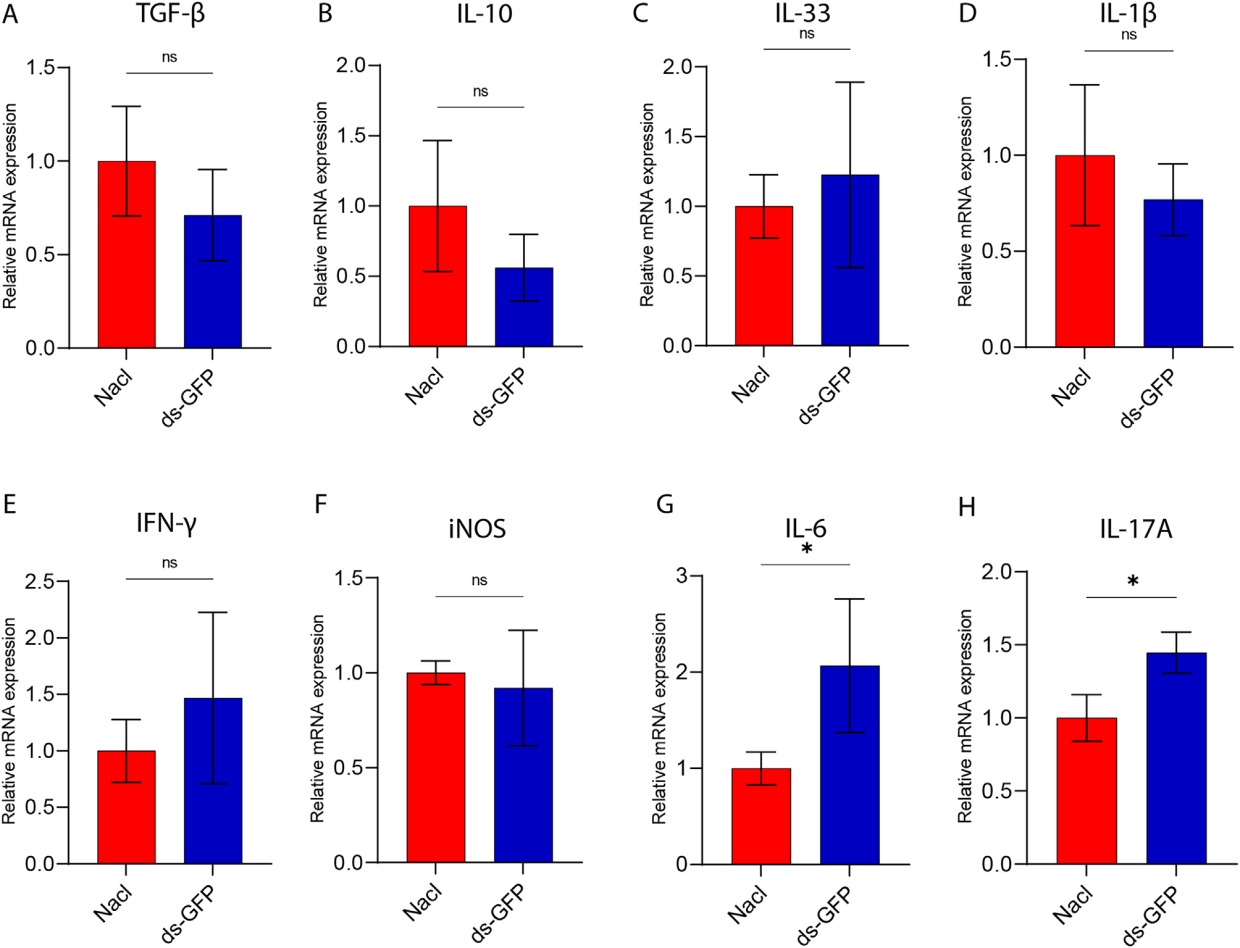
RT-qPCR analysis of proinflammatory genes in mice spleen treated with ds-GFP or NaCl. Eight mice were randomly divided into the NaCl group and the ds-GFP group, with each mouse being infected percutaneously with 60 ± 2 cercariae. The mice were euthanized 28 days after NaCl or ds-GFP treatment. The spleen was collected and total RNA was extracted by TRIzol reagent. The relative mRNA expression of TGF-β (A), IL-10 (B), IL-33 (C), IL-1β (D), IFN-γ (E), iNOS (F), IL-6 (G), and IL-17A (H) in the spleen was detected by qPCR. *p < 0.05, ns *p* > 0.05 by unpaired *t*-test; *n* = 4. Error bars represent the SEM.

**Figure S2.**
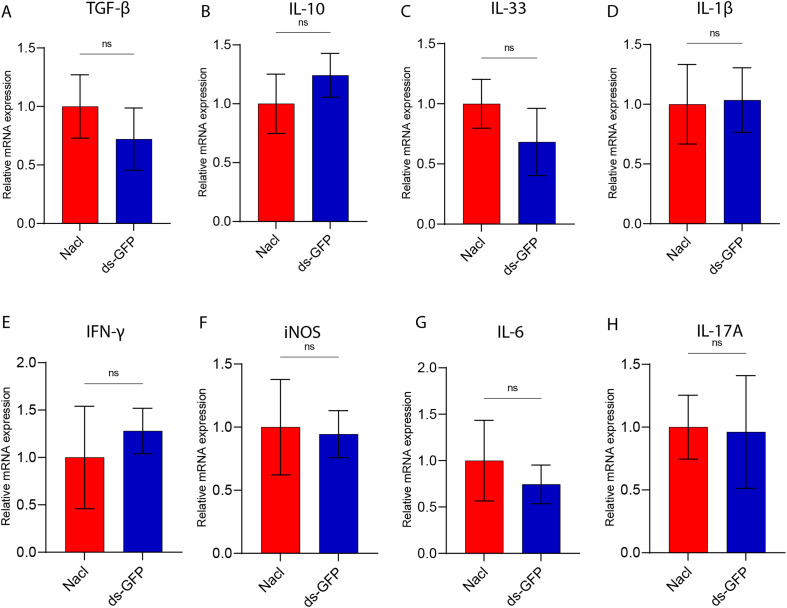
RT-qPCR analysis of proinflammatory genes in lung of mice treated with ds-GFP or NaCl. Eight mice were randomly divided into the NaCl group and the ds-GFP group, with each mouse being infected percutaneously with 60 ± 2 cercariae. The mice were euthanized 28 days after NaCl or ds-GFP treatment. The lung was collected and total RNA was extracted by TRIzol reagent. The relative mRNA expression of TGF-β (A), IL-10 (B), IL-33 (C), IL-1β (D), IFN-γ (E), iNOS (F), IL-6 (G), and IL-17A (H) in the spleen was detected by qPCR. ns *p* > 0.05 by unpaired *t*-test. *n* = 4. Error bars represent the SEM.

**Figure S3.**
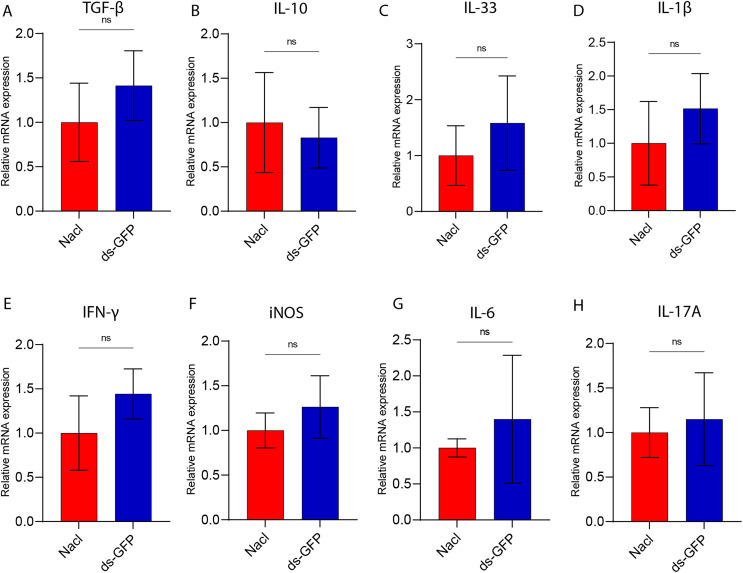
RT-qPCR analysis of proinflammatory genes in heart of mice treated with ds-GFP or NaCl. Eight mice were randomly divided into the NaCl group and the ds-GFP group, with each mouse being infected percutaneously with 60 ± 2 cercariae. The mice were euthanized 28 days after NaCl or ds-GFP treatment. The heart was collected and total RNA was extracted by TRIzol reagent. The relative mRNA expression of TGF-β (A), IL-10 (B), IL-33 (C), IL-1β (D), IFN-γ (E), iNOS (F), IL-6 (G), and IL-17A (H) in the spleen was detected by qPCR. ns *p* > 0.05 by unpaired *t*-test. *n* = 4. Error bars represent the SEM.

**Figure S4.**
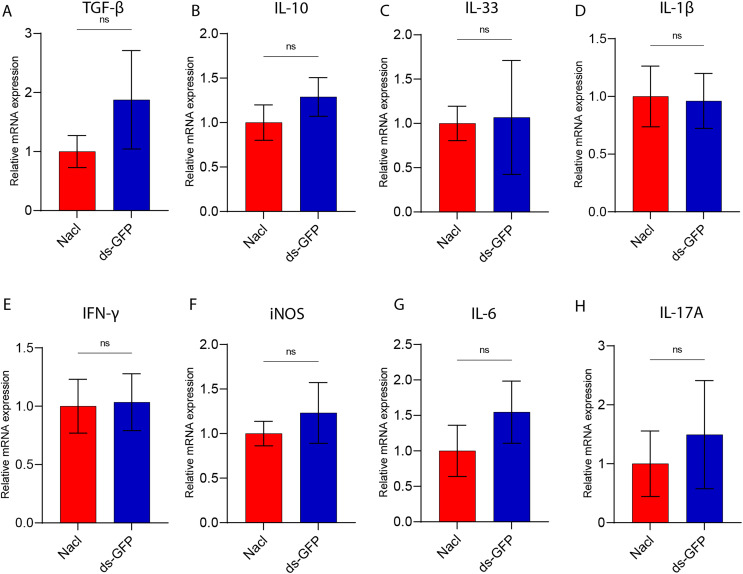
RT-qPCR analysis of proinflammatory genes in kidney of mice treated with ds-GFP or NaCl. Eight mice were randomly divided into the NaCl group and the ds-GFP group, with each mouse being infected percutaneously with 60 ± 2 cercariae. The mice were euthanized 28 days after NaCl or ds-GFP treatment. The kidney was collected and total RNA was extracted by TRIzol reagent. The relative mRNA expression of TGF-β (A), IL-10 (B), IL-33 (C), IL-1β (D), IFN-γ (E), iNOS (F), IL-6 (G), and IL-17A (H) in the spleen was detected by qPCR. ns *p* > 0.05 by unpaired *t*-test. *n* = 4. Error bars represent the SEM.

**Figure S5.**
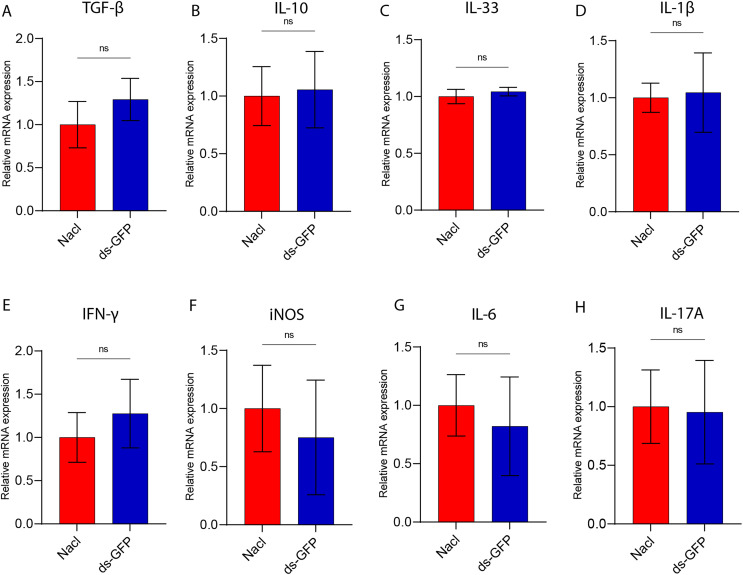
RT-qPCR analysis of proinflammatory genes in liver of mice treated with ds-GFP or NaCl. Eight mice were randomly divided into the NaCl group and the ds-GFP group, with each mouse being infected percutaneously with 60 ± 2 cercariae. The mice were euthanized 28 days after NaCl or ds-GFP treatment. The liver was collected and total RNA was extracted by TRIzol reagent. The relative mRNA expression of TGF-β (A), IL-10 (B), IL-33 (C), IL-1β (D), IFN-γ (E), iNOS (F), IL-6 (G), and IL-17A (H) in the spleen was detected by qPCR. ns *p* > 0.05 by unpaired *t*-test. *n* = 4. Error bars represent the SEM.

Furthermore, proinflammatory gene expression was assessed in various organs of mice at 28 dpi following treatment with either ds-GFP or NaCl. Notably, in the spleen of ds-GFP-treated mice, the levels of the inflammatory cytokines IL-6 and IL-17A were found to be significantly elevated compared to those in NaCl-treated mice (Figs. 3G, 3H). Conversely, the expression of TGF-β, IL-10, IL-33, IL-1β, IFN-γ, and iNOS in the spleen showed no significant differences between ds-GFP-treated and NaCl-treated mice (Figs. 3A–3F). Similarly, there were no substantial alterations in the proinflammatory gene profiles of the lung, heart, kidney, and liver in mice treated with ds-GFP or NaCl at 28 dpi (Figs. S2–S5). Taken together, these findings suggest that ds-GFP treatment had a minimal impact on body and organ weights, but did lead to some immune responses, particularly an increase in certain cytokines at 28 dpi.

### The administration of ds-GFP does not provoke significant organ damage in mice

**Figure 4:**
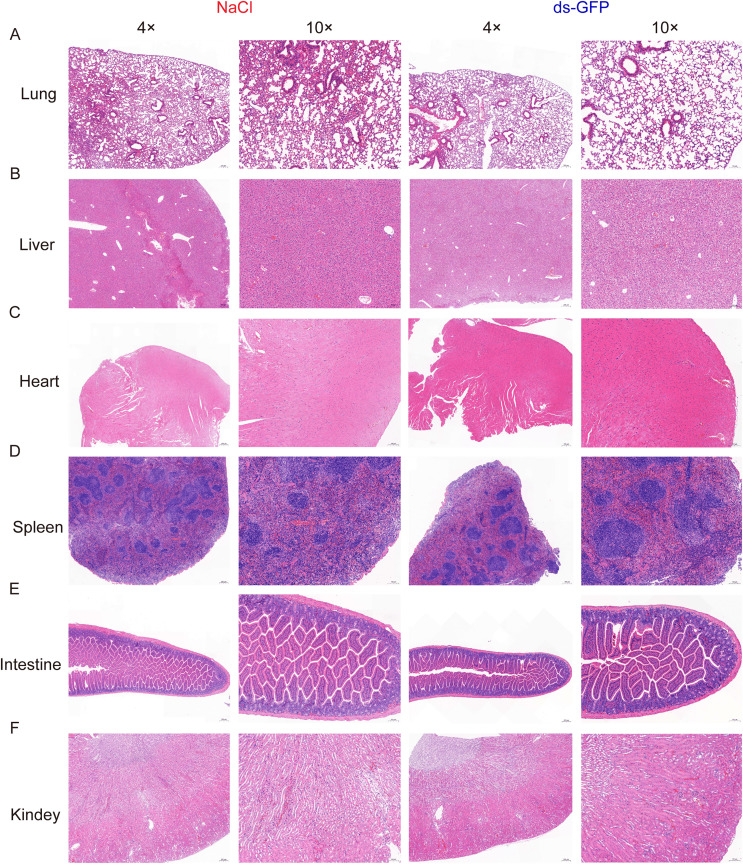
No significant organ damage in mice following administration of ds-GFP. Eight mice were randomly divided into the NaCl group and the ds-GFP group, with each mouse being infected percutaneously with 60 ± 2 cercariae. The mice were euthanized 28 days after NaCl or ds-GFP treatment. H&E staining of: (A) lung tissue; (B) liver; (C) heart; (D) spleen tissue; (E) intestine; and (F) kidney; *n* = 4.

To further evaluate potential adverse effects on organ damage in mice, additional comprehensive assessments were conducted. In-depth histopathological examinations were conducted on liver, kidney, heart, lung, intestinal, and spleen tissues at 28 days post-treatment with either ds-GFP or NaCl. Histological findings consistently reinforced the absence of significant alterations in tissue architecture or cellular morphology following ds-GFP administration. In the liver, there were no indications of hepatocellular necrosis, inflammation, or fibrosis in mice treated with ds-GFP (Fig. 4B). Similarly, the kidneys from ds-GFP treated mice exhibited normal glomerular and tubular structures without signs of nephrotoxicity (Fig. 4F). The cardiac tissue displayed regular myocardial architecture, and the pulmonary parenchyma showed no evidence of inflammation or fibrotic changes compared to the control group (Figs. 4A, 4C). Additionally, spleen sections revealed unremarkable histology with intact white and red pulp structures in the ds-GFP treated mice (Fig. 4D). Microscopic examination of intestinal tissues consistently revealed normal histological features in both the small and large intestines of ds-GFP-treated mice. The villi and crypt structures in the small intestine appeared well-preserved, and there were no signs of epithelial damage, inflammation, or alterations in goblet cell distribution (Fig. 4E). This suggests that even prolonged exposure to ds-GFP does not pose a significant risk to overall organ health.

### The immune cell profile in mice was unaffected following ds-GFP administration

**Figure 5:**
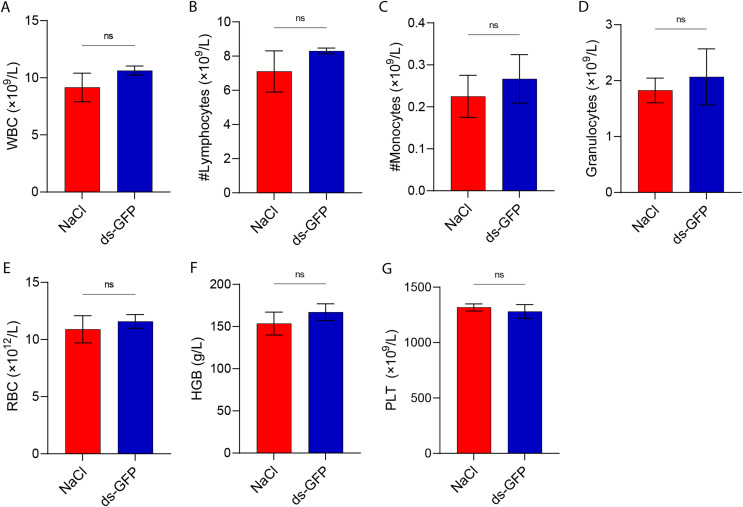
Whole blood cell analysis of ds-GFP or NaCl-treated mice. Eight mice were randomly divided into the NaCl group and the ds-GFP group, with each mouse being infected percutaneously with 60 ± 2 cercariae. The mice were euthanized 28 days after NaCl or ds-GFP treatment. The blood samples of mice were collected and prepared for anticoagulant blood. The levels of white blood cells (WBC) (A), lymphocytes (LYM) (B), monocytes (MONO) (C), granulocytes (GRAN) (D), red blood cells (RBC) (E), hemoglobin (HGB) (F), and platelets (PLT) (G) were determined using an automatic blood analyzer. ns *p* > 0.05 by unpaired *t*-test; *n* = 4. Error bars represent the SEM.

To further investigate the impact of ds-GFP on peripheral immune cells, mice were anesthetized at 28 dpi following treatment with either ds-GFP or NaCl. Blood samples were collected from the fundus venous plexus for subsequent blood routine analysis. Analysis of white blood cells (WBC) revealed a slight increase in lymphocytes, monocytes, and granulocytes in the peripheral blood of ds-GFP-treated mice; however, these differences did not reach statistical significance when compared to the control group (Figs. 5A–5D). Furthermore, levels of red blood cells (RBC), hemoglobin (HGB), and platelets (PLT) showed no significant variations between ds-GFP-treated and NaCl-treated mice (Figs. 5E–5G).

**Figure 6:**
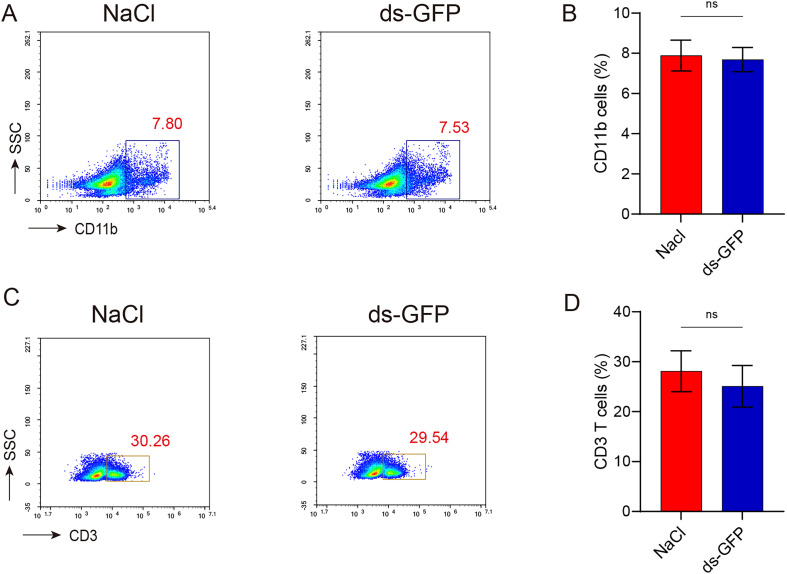
Proportion of immune cells in mice spleen not affected by ds-GFP. Eight mice were randomly divided into the NaCl group and the ds-GFP group, with each mouse being infected percutaneously with 60 ± 2 cercariae. The mice were euthanized 28 days after NaCl or ds-GFP treatment. Lymphocytes from spleen of mice were pooled and nylon-wool purified and CD11b and CD3 cells were stained for flow cytometric analysis. (A) Representative flow plots of CD11b cells in spleen at 28 days after NaCl or ds-GFP treatment. (B) Frequency of CD11b cells. (C) Representative flow plots of CD3 T cells in spleen at 28 days after NaCl or ds-GFP treatment. (D) Frequency of CD3 T cells. ns *p* > 0.05 by unpaired *t*-test; *n* = 4. Error bars represent the SEM.

Additionally, in order to assess the impact of ds-GFP on the proportion of immune cells in mice spleen, flow cytometry analysis was conducted based on the outcomes of routine blood analysis. CD3 and CD11b were specifically chosen for this purpose. Notably, no significant alterations were observed in the proportions of CD11b^+^ and CD3^+^ cells among both ds-GFP-treated and NaCl-treated mice (Figs. 6A–6D). Overall, it can be concluded that ds-GFP did not exert a substantial influence on the immune response of mice.

### ds-GFP induces gene expression alterations in mice spleen

**Figure 7:**
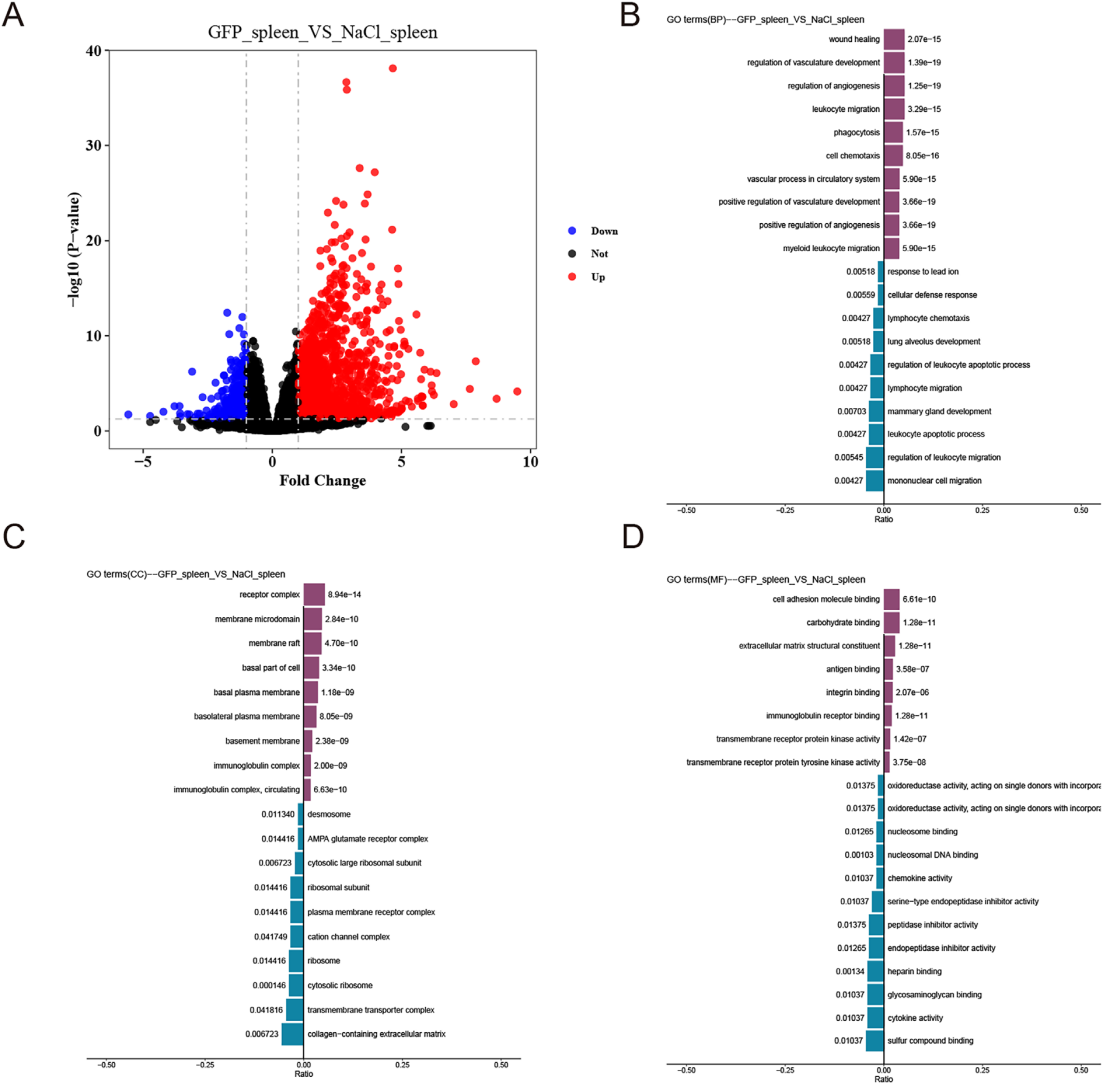
Gene expression in mice spleen altered by ds-GFP treatment. RNA-Seq was performed on mice spleen at 28 days after NaCl or ds-GFP treatment. (A) Volcano plots showing differentially expressed genes (DEGs) in ds-GFP compared to NaCl. (B) Gene Ontology (GO) analysis for biological process in DEGs (*p* < 0.05). (C) Gene Ontology (GO) analysis for cellular components in DEGs (*p* < 0.05). (D) Gene Ontology (GO) analysis for molecular functions in DEGs (*p* < 0.05).

The investigation delved into the molecular realm, specifically examining alterations in gene expression within mice spleen following ds-GFP administration. The contrast between ds-GFP-treated and NaCl-treated groups showed that ds-GFP challenge induced more upregulated DEGs than downregulated DEGs, with a false discovery rate (FDR) less than 0.05 at 28 dpi (Fig. 7A and Supplementary Table 2). To ensure a comprehensive understanding of the functional implications of differentially expressed genes (DEGs), gene ontology (GO) analysis was conducted to categorize and annotate these genes based on biological processes (BP), cellular components (CC), and molecular functions (MF). In the spleen at 28 days dpi, the GO analysis revealed notable trends in biological processes. “Wound healing”, “angiogenesis”, and “phagocytosis” were identified as significantly up-regulated processes in response to ds-GFP challenge (Fig. 7B). These findings suggest that ds-GFP may contribute to enhanced tissue repair, vascular development, and phagocytic activity within the spleen. Conversely, immune response-related biological processes were observed to be down-regulated by ds-GFP. This down-regulation may indicate a modulation of immune-related pathways, potentially reflecting a regulatory influence of ds-GFP on specific components of the immune system in the spleen (Fig. 7B). At the cellular component level, the GO analysis revealed an upregulation of “membrane” and “immunoglobulin complex”, while “collagen-containing extracellular matrix”, “transmembrane transporter complex”, and “ribosome” were downregulated (Fig. 7C). These changes indicate potential alterations in membrane-associated structures and immune-related complexes, suggesting a dynamic impact of ds-GFP on the cellular microenvironment within the spleen. In addition, the analysis of molecular functions demonstrated that “cell adhesion molecule binding”, “carbohydrate binding”, “extracellular matrix structural constituent”, and “antigen binding” were upregulated, while “sulfur compound binding”, “cytokine and chemokine activity”, “glycosaminoglycan binding”, and “heparin binding” were downregulated by ds-GFP (Fig. 7D). These molecular function alterations point towards potential changes in cell adhesion, extracellular matrix composition, and antigen recognition within the spleen induced by ds-GFP. In summary, these results provide detailed insights into the diverse functional consequences of ds-GFP-induced gene expression alterations in the spleen.

### ds-GFP exhibited no significant impact on the growth and development of *S. japonicum*

**Figure 8:**
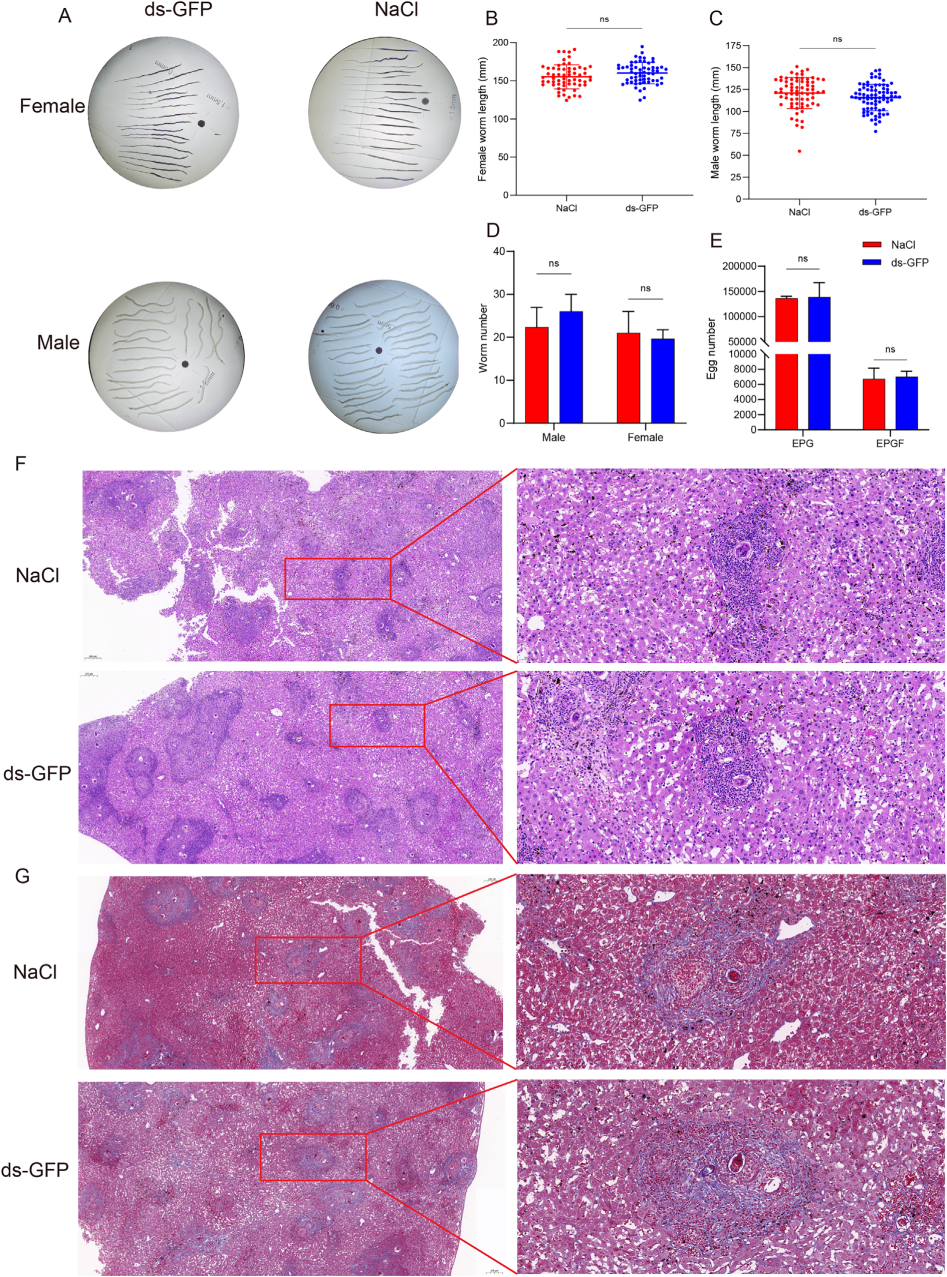
The growth and development of *S. japonicum* remained largely unaffected by ds-GFP. Adult male and female *S. japonicum* were harvested at 28 dpi. (A) Representative of female and male worm body-length between ds-GFP and NaCl. (B) Female worm body-length measurements. ns *p* > 0.05 by unpaired *t*-test; *n* > 30. (C) Male worm body-length measurements. ns *p* > 0.05 by unpaired *t*-test; *n* > 30. (D) Number of female and male worms per mouse. ns *p* > 0.05 by unpaired *t*-test; *n* = 4. (E) Number of eggs from ds-GFP or NaCl treated mice. EPG: eggs per gram; EPGF: egg counts per gram of faeces. ns *p* > 0.05 by unpaired *t*-test; *n* = 4. (F) Histological assessment of mouse liver by staining. (G) Histological assessment of mouse liver by Masson staining. Each group had four biological replicates, the images shown were representative experimental results of one mouse. Error bars represent the SEM.

In tandem with the investigation into the effects of ds-GFP on the host immune system, particular attention was directed toward its impact on the growth and development of *S. japonicum*. Morphological assessments and quantitative measurements of *S. japonicum* specimens did not reveal any noteworthy deviations or disruptions attributable to ds-GFP treatment (Figs. 8A–8D). The parasites exhibited typical development patterns, and their overall health and vitality remained comparable to those observed in the control group. Notably, our analyses revealed that ds-GFP administration did not result in a significant change in the number of *S. japonicum* eggs compared to the control group (Fig. 8E). Both groups exhibited similar egg counts, suggesting that ds-GFP did not interfere with the reproductive capacity of the parasites within the host tissues. In addition, liver sections from both ds-GFP-treated and control mice displayed characteristic granuloma structures (Fig. 8F). While there were no discernible differences in the size and distribution of granulomas between the two groups, which may suggest that ds-GFP did not significantly alter the host’s granulomatous response to *S. japonicum* eggs, it is important to acknowledge that these conclusions are drawn solely from histological observations. Importantly, both ds-GFP-treated and control groups exhibited similar patterns of collagen distribution within granulomas, indicating that ds-GFP did not appear to exacerbate or attenuate fibrotic responses in the liver (Fig. 8G). Overall, these findings, based on histological evidence, suggest that ds-GFP treatment had no notable adverse effects on the growth, development, or reproductive capacity of *S. japonicum*, and may not significantly impact the host’s immune and fibrotic responses.

**Figure 9:**
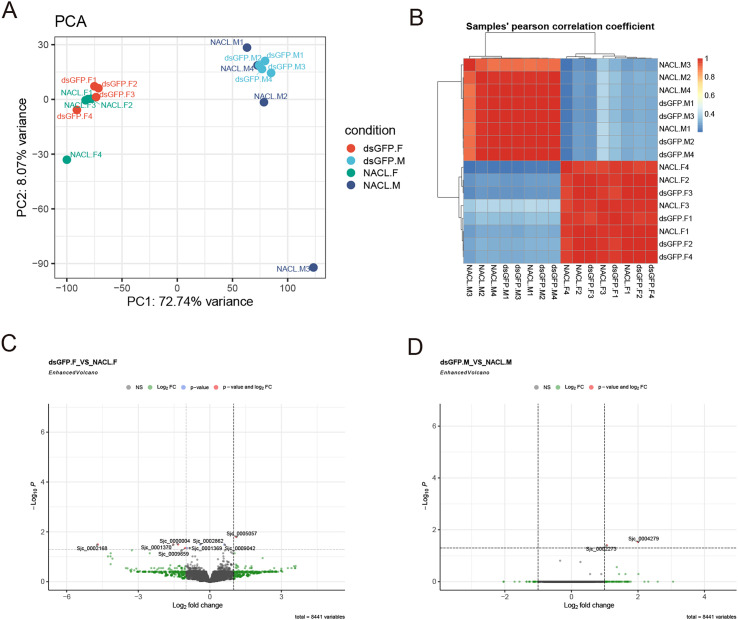
Differentially expressed genes (DEGs) of *S. japonicum* after ds-GFP RNAi *in vivo.* Adult male and female *S. japonicum* were harvested at 28 dpi and RNA-Seq was performed on replicate samples of dsGFP.F (female *S. japonicum* worms treated with ds-GFP), dsGFP.M (male *S. japonicum* worms treated with ds-GFP), NACL.F (female *S. japonicum* worms treated with NaCl), and NACL.M (male *S. japonicum* worms treated with NaCl). (A) PCA results. Each symbol indicates an individual sample. (B) Pearson’s correlation analysis between samples. (C) Volcano plots showing differentially expressed genes (DEGs) in ds-GFP.F compared to NaCl.F. (D) Volcano plots showing differentially expressed genes (DEGs) in ds-GFP.M compared to NaCl.M.

To further explore the biological responses of ds-GFP in the development of *S. japonicum*, we performed RNA-seq analysis of both adult male and female worms *in vivo*. As shown in Figure 9A, the Principal Component Analysis (PCA) results demonstrate that the patterns exhibited by ds-GFP.F (female *S. japonicum* worms treated with ds-GFP) and NACL.F (female *S. japonicum* worms treated with NaCl) are consistent, as are those of ds-GFP.M (male *S. japonicum* worms treated with ds-GFP) and NACL.M (male *S. japonicum* worms treated with NaCl). Additionally, the Pearson correlation analysis showed that these 16 RNA-seq samples were well clustered by gender rather than ds-GFP treatment (Fig. 9B). We then evaluated differential gene expression in both male and female *S. japonicum* after ds-GFP RNAi. Of a total of 8,441 variables, 1 was up-regulated and 4 were down-regulated in ds-GFP.F *vs* the NACL.F group (Fig. 9C). In addition, only 2 genes were up-regulated in ds-GFP.M *vs* the NACL.M group (Fig. 9D). Taken together, these findings highlight that ds-GFP did not significantly impact the gene expression pattern in *S. japonicum*.

## Discussion

RNAi has emerged as a preferred tool in parasite research for investigating gene function, capitalizing on the intrinsic RNAi machinery of these organisms. dsRNA, in particular, has proven to be a simple, yet essential method for studying gene function in schistosomes and other parasites [5, 18, 20]. However, the potential off-target effects of dsRNAs, demonstrated across various biological systems, raise concerns, especially with high concentrations that may saturate the RISC complex, leading to systemic dysregulation of gene expression [2, 9, 13, 17, 24]. Given that GFP is commonly used as a control in *S. japonicum* RNAi experiments, the potential for altered expression of off-target genes in other species should be considered [16]. The primary aim of our study was to reevaluate the safety and suitability of ds-GFP as an RNAi control in *S. japonicum*. In this study, we confirmed that ds-GFP is a safe RNAi control with minimal impact on host physiology and immune responses. The comprehensive assessments demonstrated no significant organ damage in mice and no adverse effects on the growth or reproductive capacity of the parasitic organism. These findings enhance the reliability of ds-GFP as a suitable control in experimental designs involving *S. japonicum*.

In the assessment of the effects of ds-GFP on mice, our results demonstrated that ds-GFP treatment did not induce significant changes in the body and organ weights of C57BL/6 J mice over a 28-day period (Fig. 1). The weights of five major organs and the maintenance of normal food and water intake further supported the conclusion that ds-GFP did not exert a substantial influence on the overall physiological parameters of the mice. Despite these minimal impacts on body and organ weights, ds-GFP did elicit a modest splenic immune response, as evidenced by the upregulation of specific cytokines at 28 dpi (Fig. 2). Studies have reported that dsRNA can induce an undesirable activation of the interferon (IFN) response [19]. Utilizing a multiplex cytokine bead array assay, we assessed 22 cytokines/chemokines in serum samples from mice subjected to ds-GFP or NaCl treatments at days 0, 14, 21, and 28 post-injection. Consistent with previous reports, by day 28, ds-GFP-treated mice displayed significantly elevated concentrations of pro-inflammatory cytokines (IL-6, IL-17A, IL-12(p70), IFN-γ, MIP-β), and notably, anti-inflammatory cytokine IL-10 levels were also significantly increased compared to NaCl-treated mice. Encouragingly, there were no significant changes observed in the levels of the remaining 11 chemokines and cytokines (Eotaxin, KC, MIP-1α, MCP-1, G-CSF, GM-CSF, IL-12(p40), IL-13, IL-1α, IL-3, IL-4, IL-5, IL-9, and TNF-α) in both ds-GFP-treated and NaCl-treated mice (Fig. 2 and Fig. S1). These findings indicate that ds-GFP treatment led to an increase in several pro-inflammatory and anti-inflammatory cytokines at 28 dpi. This immune response was further corroborated by the upregulation of inflammatory cytokines, particularly IL-6 and IL-17A, in the spleen of ds-GFP-treated mice (Figs. 3G, 3H). However, these alterations did not result in significant changes in the peripheral blood immune cell profile, as evidenced by routine blood analysis and flow cytometry (Figs. 4 and 5).

In the molecular realm, RNA-seq analysis of the spleen revealed notable gene expression changes induced by ds-GFP treatment. The PCA plot showed clear separation between ds-GFP-treated and NaCl-treated groups, and GO analysis indicated changes in biological processes related to wound healing, angiogenesis, and phagocytosis. Additionally, changes in cellular components and molecular functions suggested potential impacts on cell adhesion, extracellular matrix composition, and antigen recognition within the spleen, induced by ds-GFP (Fig. 7). Importantly, histopathological examinations of multiple organs, including the liver, kidneys, heart, lungs, intestines, and spleen, at 28 dpi showed no signs of significant damage or abnormalities following ds-GFP administration (Fig. 6). These comprehensive assessments collectively suggest that ds-GFP treatment had minimal impact on body and organ weights, but did induce some immune responses in mice, particularly an increase in certain cytokines at 28 dpi. Notably, these alterations did not adversely affect the overall health and vitality of the mice.

Moving beyond host immune response, our study also investigated the impact of ds-GFP on *S. japonicum*, the parasitic organism. Morphological assessments and quantitative measurements of *S. japonicum* specimens revealed no significant deviations or disruptions attributable to ds-GFP treatment (Figs. 8A–8D). The reproductive capacity of the parasites, as indicated by egg counts, remained unaffected by ds-GFP (Fig. 8E). Furthermore, liver sections from ds-GFP-treated mice displayed typical granuloma structures and collagen distribution patterns comparable to those in the control group, indicating that ds-GFP did not significantly alter the host’s granulomatous and fibrotic responses to *S. japonicum* eggs (Fig. 8F).

In summary, employing meticulous primer design and rigorous techniques, particularly *in vivo* experiments with mice infected by *S. japonicum* cercariae, we established that ds-GFP treatment did not result in a significant loss in mouse body weight. While a splenic immune response was found, indicated by the elevation of cytokines at 28 dpi, it is interesting that the immune cell profile in mice remained unaffected following ds-GFP administration. Additionally, administration of ds-GFP did not induce significant organ damage and had no adverse effects on the growth, development, or reproductive capacity of *S. japonicum*. This comprehensive evaluation provides substantial evidence supporting the safety of ds-GFP as an RNAi control in the context of *S. japonicum* studies, thereby endorsing its reliability in facilitating accurate research outcomes in studying schistosomiasis and related parasitic diseases.

## Data Availability

The raw sequencing data generated in this study have been submitted to the NCBI Sequence Read Archive (SRA) database under accession number PRJNA1100745 and can be accessed via this link: https://www.ncbi.nlm.nih.gov/bioproject/PRJNA1100745.

## Supplementary material

The supplementary material for this article can be found at https://www.parasite-journal.org/10.1051/parasite/2025003/olm.

*Supplementary Table 1*. All primers for this study. *Supplementary Table 2*. GFP_spleen_VS_NaCl_spleen_DEGenes and GFP_spleen_VS_NaCl_spleen_GOterms_enrichment. *Supplementary Table 3*. dsGFP.F_VS_NACL.F_DEGenes and dsGFP.M_VS_NACL.M_DEGenes.
